# Site-specific recombinatorics: in situ cellular barcoding with the Cre Lox system

**DOI:** 10.1186/s12918-016-0290-3

**Published:** 2016-06-30

**Authors:** Tom S. Weber, Mark Dukes, Denise C. Miles, Stefan P. Glaser, Shalin H. Naik, Ken R. Duffy

**Affiliations:** Hamilton Institute, Maynooth University, Maynooth, Ireland; University of Strathclyde, Glasgow, UK; The Walter and Eliza Hall Institute of Medical Research & The University of Melbourne, Parkville, Melbourne, Australia

**Keywords:** Cell fate tracking, Cellular barcoding, Cre lox system, DNA stochastic programme, Combinatorial explosion

## Abstract

**Background:**

Cellular barcoding is a recently developed biotechnology tool that enables the familial identification of progeny of individual cells in vivo. In immunology, it has been used to track the burst-sizes of multiple distinct responding T cells over several adaptive immune responses. In the study of hematopoiesis, it revealed fate heterogeneity amongst phenotypically identical multipotent cells. Most existing approaches rely on ex vivo viral transduction of cells with barcodes followed by adoptive transfer into an animal, which works well for some systems, but precludes barcoding cells in their native environment such as those inside solid tissues.

**Results:**

With a view to overcoming this limitation, we propose a new design for a genetic barcoding construct based on the Cre Lox system that induces randomly created stable barcodes in cells in situ by exploiting inherent sequence distance constraints during site-specific recombination. We identify the cassette whose provably maximal code diversity is several orders of magnitude higher than what is attainable with previously considered Cre Lox barcoding approaches, exceeding the number of lymphocytes or hematopoietic progenitor cells in mice.

**Conclusions:**

Its high diversity and in situ applicability, make the proposed Cre Lox based tagging system suitable for whole tissue or even whole animal barcoding. Moreover, it can be built using established technology.

**Electronic supplementary material:**

The online version of this article (doi:10.1186/s12918-016-0290-3) contains supplementary material, which is available to authorized users.

## Background

The fate of the progeny of two seemingly identical cells can be markedly distinct. Well studied examples include the immune system and hematopoietic system, for which the extent of clonal expansion and differentiation has been shown to vary greatly between cells of the same phenotype [1–4]. Fate and expression heterogeneity at the single-cell level are also apparent in other systems including the brain [5–7] and cancers [8–10]. Whether this heterogeneity is due to the stochastic nature of cellular decision making, reflects limitations in phenotyping, is caused by external events, or a mixture of effects, is a subject of active study in several fields [11–13]. As addressing this pivotal question through population-level analysis is not possible, experimental tools have been developed that facilitate monitoring single cells and their offspring over several generations.

Long-term fluorescence microscopy represents the most direct approach to assess fate heterogeneity at the single-cell level. Studies employing that technique are numerous [14–20], and have revealed, among many other significant observations, that although the fate of stimulated B cells are heterogeneous, there exist strong correlations at the clonal level in terms of differentiation and death versus division fates [15, 21]. Filming and tracking of cell families in vitro remains technically challenging, is labor intensive, and only partially automatable [22, 23]. Despite significant advances in the field, continuous tracking in vivo is confined to certain tissues, and time windows of up to twelve hours for slowly or non-migrating cells.

A radically different approach to long-term clonal monitoring is to mark single cells with unique DNA tags via retroviral transduction, a technique known as cellular barcoding [2, 10, 24–27]. As tags are heritable, clonally related cells can be identified via DNA sequencing. By tagging multi-potent cells of the hematopoietic system and adoptively transferring them into irradiated mice, the contribution of single stem cells to overall hematopoiesis has been quantified [24–26, 28]. Amongst other discoveries, this has revealed statistically consistent heterogeneity in the collection of distinct cell types produced from apparently equi-potent progenitors [29–31]. Current barcoding techniques are unsuitable for tagging cells in vivo, and typically require ex vivo barcoding followed by adoptive cell transfer [26]. This restricts its scope to cells suitable for adoptive transfer, such as hematopietic stem and progenitors, naive lymphocytes, and cancer cells.

Ideally, a cellular barcoding system would inducibly mark cells in situ in their native environment, would be non-toxic, permanent and heritable, barcodes would be easy to read with a high-throughput technique, and the system would enable labeling large numbers of cells with unique barcodes. Recently, two studies have been published that address some of these points. Sun et al. [32] employed a Dox inducible hyperactive form of the Sleeping Beauty transposase to genetically tag stem cells in situ, and followed clonal dynamics during native hematopoiesis in mice. In that system, tags consist of a random insertion site of an artificial transposon, which upon withdrawal of Dox is relatively stable. A second in vivo cellular barcoding system based on site-specific DNA recombination with the Rci invertase was implemented by Peikon and co-workers [33–35]. Inspired by the brainbow mouse [36], this system induces a random barcode by stochastically shuffling a synthetic cassette pre-integrated into the genome of a cell. The authors predicted high code diversity from relatively small constructs (approx. 2 kb) and demonstrated feasibility of random barcode generation in Escherichia coli [35].

Each of those approaches provide elegant advances on shortcomings of previous systems by generating largely unique tags without significant perturbation to the system of interest, but some difficulties remain. For barcode readout, the method in [32] requires whole-genome amplification technology and three arm-ligation-mediated PCR to efficiently amplify unknown insertion sites. Furthermore, the random location of the transposon may impact behavior of some barcoded clones and thus lead to biased data. Moreover, some background transposon mobilization was detected in certain cell types, subverting the stability of the barcodes. The Rci invertase based system remains to be implemented in cells other than bacteria. Similar to the Sleeping Beauty transposase, the method requires tight temporal control over Rci expression to make codes permanent.

In the present article, we consider the Cre Lox system as a driver to induce in situ from a series of tightly spaced Lox sites large numbers of distinct, permanent, randomly determined barcodes. In contrast to the Brainbow construct [36, 37], which relies on overlapping pairs of incompatible Lox sites to recombine randomly into one of several stable DNA sequence configurations, our design exploits constraints on the distance between Lox sites that arise during DNA loop formation, a prerequisite for site-specific recombination [38–40]. This known feature has not previously been exploited, but is a crucial design element for obtaining high barcode diversity. First, by allowing repeated usage of the same Lox site, code diversity is solely restricted by cassette size and not, as in the Brainbow construct, by the relatively small set of non-interacting Lox sites [41]. Second, for a design without distance constraints, the diversity of stable barcodes creatable with the Cre Lox system is of order *O*(*n*) at best, where *n* is the number of Lox sites [35]. Whereas with distance contraints, optimal barcode diversities of order *O*(*n*^3^) are possible. As will be shown in this article, boosting this scaling with the four incompatible Lox sites that have been reported in the literature [41], 10^12^ distinct codes of about 600 bp each can be generated from a genetic construct as small as 2.5 kb. In combination with the CreEr system [42], this is sufficient to inducibly barcode label e.g. all naive CD8 T cells in a mouse [43]. Desirable features are inherently part of the Lox barcode cassette design, including: short and stable barcodes; a single barcode per cell; and robust read-out.

### Cre Lox biology

**Fig. 1:**
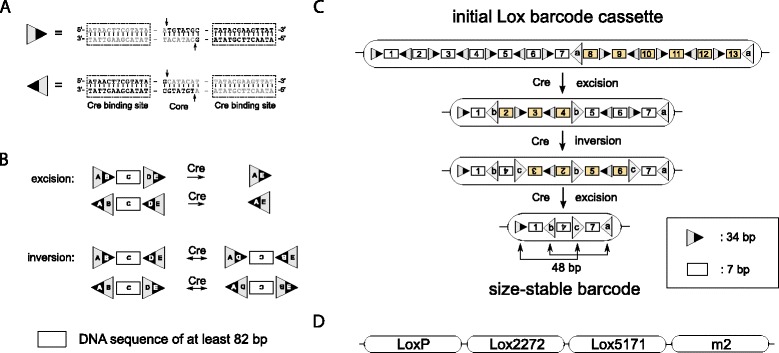
Lox biology and Lox barcode cassette. **a** Lox DNA sequences. Lox sites are composed of two 13 bp palindromic Cre binding sites and an 8 bp core (original LoxP sequence shown). Asymmetric cleavage sites in the core are indicated by arrows. **b** Cre mediated site-specific excision and inversion of a sequence with a minimum of 82 bp between two Lox sites on the same chromosome [38]. If Lox sites are oriented in the same direction, productive recombination excises the sequence, while if they are oriented in opposite direction the sequence is inverted (i.e., the reverse complement). **c** An alternating Lox cassette with 13 elements of size 7 bp. To illustrate how barcodes are generated, two excision and one inversion event are shown that create from the initial cassette a size-stable barcode with three random elements. Pairs of interacting Lox sites are indicated by a, b, and c. Elements affected by recombination have colored background. The barcode with three elements is size-stable as Lox sites oriented in the same direction (*arrows*) are closer than the minimal Lox interaction distance of 82 bp, precluding further excision. **d** Four concatenated alternating Lox cassettes of length 13 elements each with poorly-interacting Lox site variants [41] result in a code diversity greater than 10^12^

Before introducing the Lox barcode cassette, we revisit some facts about Cre Lox biology [44, 45]. Cre is a bacteriophage Pl recombinase that catalyzes site-specific recombination between Lox sites. A Lox site is a 34 bp long sequence composed of two 13 bp palindromic flanking regions and an asymmetric 8 bp core region (Fig. 1a). For recombination to occur four Cre proteins bind to the four palindromic regions of two Lox sites and form a synaptic complex. A first pair of strand exchanges leads to a Holliday junction intermediate [46]. Isomerization of the intermediate then allows a second pair of strand exchanges, and formation of the final recombinant product [40]. The DNA cleavage site is situated in the asymmetric core region. If the Lox sites are on the same chromosome, their interaction requires formation of a DNA loop. If they have the same orientation (direct repeats), recombination results in excision of the intervening sequence, and this reaction is essentially unidirectional [47]. If Lox sites are in the opposite orientation to each other (inverted repeats), the sequence between the sites is inverted, becoming its reverse complement (Fig. 1b). In the absence of Cre, Lox-Lox recombination events are below detection limits (e.g. [37], Fig. S1). Due to compatibility with eukaryotes, the Cre Lox system has become an essential tool in genetic engineering and a large array of transgenic mouse models with inducible cell-type specific expression of Cre have been created [42].

In in vitro trials with Cre mediated Lox reactions, a sharp decrease in recombination efficiency has been observed when the sequence separating two Lox sites is less than 94 bp [38]. Recombination is still detectable at low levels at 82 bp, but not at 80 bp where DNA stiffness appears to prevent DNA loop formation, and as a consequence Lox site interaction. For the distinct, but similar, Flp/FRT system this minimal distance was established to be smaller in vivo, with interactions still possible at 74 bp [39]. The existence of a minimal distance is one of the key features that we will exploit to make random barcodes stable, but in our proposed design it will only prove necessary for it to be greater than 44 bp.

### Lox barcode cassettes

In full generality, a Lox barcode cassette is a series of Lox sites interlaced with *n* distinguishable DNA code elements of size *m* bp each. On Cre expression, code elements change orientation and position, or are excised [34]. Through Cre mediated excision, the number of elements eventually decreases until reaching a stable number (Fig. 1c). Sequences that have attained a stable number of code elements form size-stable barcodes. A cassette’s code diversity is the number of size-stable barcodes that can be generated from the cassette via site-specific recombination.

Our main result is a robust Lox cassette design that provably maximizes code diversity for any given cassette length *n* and element length *m*≥5 bp. The design is robust to both sequencing errors and to the minimal interaction distance between Lox sites. The analysis that leads us to the design is provided in the “Optimal design” Section. The identification of code element sequences that avoid misclassification due to sequencing mismatch errors then follows. Finally, probabilistic aspects of code generation from an optimal barcode cassette are explored via Monte Carlo simulation. Lox cassettes with code elements of size 4 bp, higher order Lox interactions, the impact of transient Cre activation, and distance-dependent Lox-Lox complex formation are considered in the discussion.

## Results

### A robust cassette design that maximizes code diversity

The optimal design will prove to have the orientation of both the outmost, and any two consecutive, Lox sites inverted (Fig. 1c). Code elements between Lox sites are of size longer than four bp, but shorter than 24 bp. The lower limit ensures that elements can be chosen sufficiently distinctly to correct for at least two sequencing errors per element. Due to the minimal Lox interaction distance, the upper limit is necessary to ensure that barcodes with three code elements are size-stable.

The barcode diversity for this cassette design with *n* code elements under constitutive Cre expression will, as shown in the Optimal design Section, transpire to be

1 $$\begin{array}{*{20}l} \frac{(n+1)(n-1)^{2}}{2}+(n+1) =O(n^{3}), \end{array} $$

which is maximal for code elements that are larger than four base pairs.

A good compromise between cassette length, robustness to sequencing errors and barcode diversity is given by an alternating Lox cassette with 13 elements of length 7 bp each as shown in Fig. 1c. The cassette is initially 567 bp long, which after excisions and inversions, generates size-stable barcodes that are composed of either a single element or three elements, with lengths 75 bp and 157 bp respectively, including remaining inactive Lox sites. This generates a code diversity of 1022 barcodes, far less than the 3×10^9^ base pairs of the mouse genome, i.e. the maximal theoretical diversity achievable by the Sleeping Beauty transposase barcoding system [32].

However, concatenating four such cassettes with poorly-interacting Lox variants (e.g. LoxP, Lox2272, Lox5171 and m2 [41], Fig. 1d) yields a size-stable code diversity of 1022^4^≈10^12^. In mice, this is sufficient to tag all CD8 T cells [43] or all nucleated cells in the bone marrow [48].

### A practical implementation

To implement Cre Lox barcoding in the mouse, one could cross mice generated from embryonic stem cells that had previously been transduced with the concatenated Lox barcoding cassettes described above (2268 bp) onto a Tamoxifen inducible cell-type specific CreEr expressing background [42]. A barcoding experiment would then be initiated by administrating Tamoxifen to the animal, which activates Cre and generation of a barcode (≤628 bp) in each cell where Cre becomes active. Some time after activation, cells of interest would be harvested and sorted for specific phenotypes, and sequenced using a next generation sequencing platform that allow read-lengths >600 bp. Cells originating from the same progenitor alive at the time of tamoxifen administration would carry the same barcode. This information would then used for inference on, for example, lineage pathways and clonal fate tracking. To identify frequent barcodes that are to be discarded in the analysis (see the Barcode distribution is heterogeneous Section), in a control experiment large numbers of cells would be harvested shortly after tamoxifen administration and sequenced.

### Optimal design

A simple upper bound on the barcode diversity of *k* elements from a cassette initially containing *n* elements is the number of possible outcomes when choosing *k* from *n* elements in arbitrary order and orientation:

$$\begin{array}{*{20}l} {n \choose k} k! 2^{k} = \frac{2^{k}n!}{(n-k)!}. \end{array} $$

Although loose, it will become clear that it captures the dominant growth, *O*(*n*^*k*^), indicating the importance of *k* in generating barcode diversity and motivating a closer look at how cassette designs influence it.

For what follows, we introduce some terminology: a cassette is alternating if the orientation of any two consecutive Lox sites is inverted (Fig. 1c); outermost Lox sites are termed flanking Lox sites; and flanking sites are direct or inverted if they have the same or opposite orientation, respectively.

#### Code diversity is determined by code element length and orientation of flanking sites

Cre recombination requires a minimal distance between the interacting Lox sites. In what follows we assume that the minimal distance for Lox interaction is 82 bp, but our results will be robust for any minimal interaction distance greater than 44 bp.

**Fig. 2:**
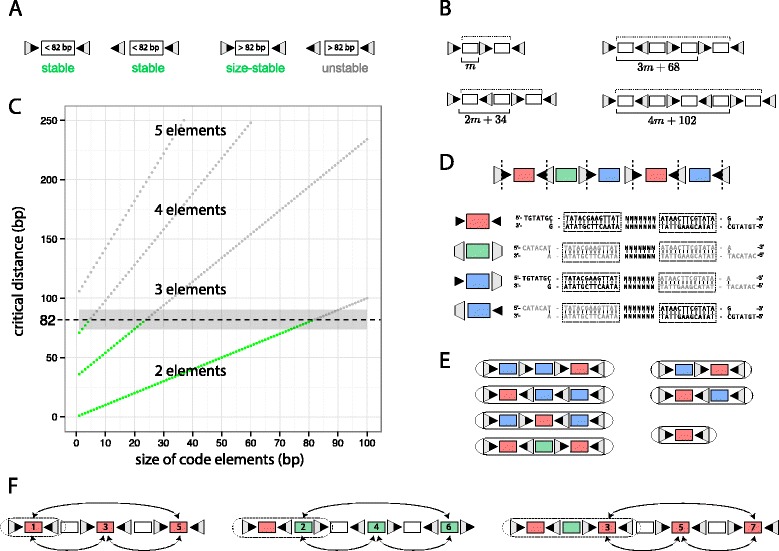
Barcode stability and code diversity. **a** The size-stability of barcodes with a single element depends on the length of the sequence between the flanking sites and their relative orientation. **b** Critical distances of barcodes of different sizes from a cassette with inverted flanking sites. The dotted line show the critical distance if flanking sites are oriented in the same direction. **c** Stability of barcodes from 2 to 5 elements for a Lox barcode cassette with inverted flanking sites. If the critical distance surpasses the minimal distance of 82 bp, stable codes (*green*) become unstable (*gray*). Barcodes of size three and four are unstable if *m*≥24 and *m*≥5 respectively, while codes of size five are always unstable. The gray interval illustrates potential uncertainty in the estimate of the minimal interaction distance. **d** Sequences between Lox cleavage sites represent the fundamental building blocks of the Lox barcode cassette. There are two with inverted Lox repeats (red, green) and two direct Lox repeats (*blue*) types of blocks. In the example, code elements are of size 7 bp and N denotes an arbitrary base. **e** For a cassette with inverted flanking sites pointing at each other and 5≤*m*<24, there are four possible block compositions ({*k*_*r*_,*k*_*g*_,*k*_*b*_}): two for barcodes of size three (three: {1,0,2} and one: {2,1,0}), one for barcodes of size two (two: {1,0,1}) and one for barcodes of size one (one: {1,0,0}). **f**) Operations (moves) on odd and even elements possible via Cre Lox recombination, in cassettes with five, six and seven elements. Each move inverts the orientation of the respective element

To understand how a minimal Lox-Lox interaction distance and cassette design determine size-stable barcodes and code diversity, we start with the simplest case, a barcode with a single code element (Fig. 2a). If the code element is less than 82 bp, the barcode is size-stable irrespective of the orientation of its flanking sites. If the element is larger than 82 bp, the code is only size-stable if the flanking sites are inverted as excision will remove the element.

For a barcode with two elements, the sequence between the flanking sites contains an additional element and a Lox site (34 bp), giving a sequence of 2*m*+34 bp. If the flanking sites have the same orientation, the barcode is size-stable if 2*m*+34<82 bp, hence if *m*<24 bp. If they are in opposite orientation, excisions can only occur if flanking sites interact with the middle Lox site, and *m*<82 bp is sufficient for stability (Fig. 2b). For given *m*, in general if there exists a barcode of size *k* with direct flanking sites, a barcode with *k*+1 elements is possible that has inverted flanking sites. Thus *m* and the orientation of the flanking sites are critical features that determine the maximum *k*.

In Fig. 2c, the stability of barcodes with *k*∈{2,3,4,5} is shown as a function of *m* for a cassette with inverted flanking sites. The stability depends on a critical distance, i.e., the largest distance between two Lox sites in the barcode that is, or can be brought into, the same orientation via recombination. As shown, barcodes of size three and four become unstable if *m*≥24 bp and *m*≥5 bp, respectively, while barcodes of size five or greater are always unstable.

Orientation of a cassette’s flanking sites is immutable under recombination. Therefore cassettes with direct and inverted flanking sites generate barcodes with direct and inverted flanking sites only. Having seen that maximal code diversity grows as *O*(*n*^*k*^), and that having inverted flanking sites relative to direct ones increases the maximum size of barcodes by one, it follows that the diversity for cassettes with inverted flanking sites is of the order *O*(*n*^*k*+1^). Inverted flanking sites are thus superior in terms of code diversity and are an essential design decision.

Optimality regarding the size of the elements, *m*, is more intricate. For *m*<5, the maximum size of barcodes is four elements, and according to the formula above, their diversity grows as *O*(*n*^4^). The stability of barcodes with four elements is, however, sensitive to the minimal distance estimate (illustrated by the gray interval in Fig. 2c). In addition, the short length of code elements limits error correction, a point revisited later. Thus we focus on cassettes in the regime 5bp≤*m*<24 bp, which generate error-robust barcodes of up to size three and a code diversity that is insensitive to the reported minimal Lox interaction distance.

#### Alternating Lox cassettes with inverted flanking sites maximize code diversity

For the orientation of the remaining Lox sites we prove, via a two-step strategy, that the alternating design produces maximal code diversity. First we derive a refined upper bound for the diversity that takes into account the structure of the Lox cassette, but ignores constraints imposed by the recombination process. We then show that alternating Lox cassettes with inverted flanking sites and *n*≥7 elements are unconstrained in terms of barcode generation via sequential recombination events, thus achieving this upper bound.

#### An upper bound for Lox barcode diversity

During Cre induced recombination, Cre proteins cleave the core region of the interacting Lox sites asymmetrically [40]. The sequences between subsequent cleavage sites are not affected by Cre and represent the fundamental building blocks of the Lox barcode cassette. Each block contains a code element and half a Lox site on each side.

Depending on the orientation of the Lox sites, there are four possible types of blocks (Fig. 2d). Three colours have been used to code these: red, green and blue. By definition, the reverse complement of a block is of the same colour class. In contrast to blue blocks, red and green blocks have their Lox cores cleaved in a way such that their flanking Lox sites are unchanged after inversion, while the intervening sequence is reverse-complemented.

Blocks are similar to the concept of units defined in [34], which proves instrumental to derive expressions for the total number of sequences, stable or unstable, that are generated from a Lox cassette where all (*n*+1) sites can interact. In our context, the latter condition implies *m*>82 and, as discussed above, a code diversity of order *O*(*n*). Here we focus on enumerating exclusively size-stable sequences that arise in the regime 5bp≤*m*<24 bp with code diversities of order *O*(*n*^3^).

Stable codes are necessarily made of blocks from the initial cassette, and as shown in Fig. 2e, their composition in terms of block colors is prescribed. Letting *n*_*r*_, *n*_*g*_, and *n*_*b*_ be the number of red, green, and blue blocks in the initial cassette with *n* elements, an upper bound on the number of possible barcodes of size *k* with *k*_*r*_ red, *k*_*g*_ green and *k*_*b*_ blue blocks is

$$\begin{array}{*{20}l} k_{r}!{n_{r}\choose{k_{r}}}k_{g}!{{n_{g}}\choose{k_{g}}}k_{b}!{{n_{b}}\choose{k_{b}}}2^{k_{r}+k_{g}}, \end{array} $$

where *n*_*r*_+*n*_*g*_+*n*_*b*_=*n* and *k*_*r*_+*k*_*g*_+*k*_*b*_=*k*. It is the number of possible outcomes when choosing *k*_*r*_, *k*_*g*_ and *k*_*b*_ from *n*_*r*_, *n*_*g*_ and *n*_*b*_ elements in arbitrary order. The additional factor $2^{k_{r}+k_{g}}\phantom {\dot {i}\!}$ arises as there are two valid orientations of every code element of a red and green block after recombination, whereas blue blocks due not enjoy this property. Conditioned on *n*_*r*_, *n*_*g*_, and *n*_*b*_, to derive an upper bound for a cassettes’s diversity, we add the numbers for the four possible stable barcode configurations of *k*_*r*_, *k*_*g*_, and *k*_*b*_, shown in Fig. 2e, taking into account that certain configurations appear more than once (e.g. the configurations with one red and two blue blocks appears three times). For 5 bp ≤*m*<24 bp, and cassettes with inverted flanking sites pointing at each other (the opposite case is similar) this yields, by applying the expression above to each of the four configurations,

$$\begin{array}{*{20}l} 3\left(1!{n_{r} \choose 1}2!{n_{b}\choose 2}\right)2^{1} +1\left(2!{{n_{r}}\choose{2}}1!{{n_{g}}\choose{1}}\right)2^{2+1}+\\ +2\left(1!{{n_{r}}\choose{1}}1!{{n_{b}}\choose{1}}\right)2^{1} +1\left(1!{{n_{r}}\choose{1}}\right)2^{1}. \end{array} $$

By construction, *n*_*g*_=*n*_*r*_−1, and since *n*_*b*_=*n*−2*n*_*r*_+1, substituting the respective terms leads to an expression that is a function of *n* and *n*_*r*_ alone. For given *n* odd, this reduces the task of finding the optimal cassette design to an explicitly solvable one-dimensional optimization problem,

$${} \begin{aligned} \underset{n_{r}}{\arg\max}\quad 32 {n_{r}^{3}} - 12 (2 n + 3) {n_{r}^{2}} + (6 n^{2} + 10 n + 14) n_{r} \quad \\\text{for}\quad n_{r} \leq \frac{n+1}{2}. \end{aligned} $$

For *n*≥5, the global maximum is achieved at the boundary *n*_*r*_=(*n*+1)/2. This implies *n*_*b*_=0, and a global upper diversity bound of (*n*+1)(*n*−1)^2^+(*n*+1), of order *O*(*n*^3^). It is easily verified that *n*_*b*_=0 is only possible if the cassette design is alternating and n is odd, which implies the flanking sites are inverted.

#### Alternating Lox cassette design achieves the upper diversity bound

For an alternating cassette design, achieving the code diversity upper bound requires complete freedom in code generation via recombination events. By construction, we show that this is the case if *n*≥7. To aid understanding, we illustrate in Fig. 2f operations on odd and even elements via Cre Lox recombination, in cassettes with five, six and seven elements. Note that each operation (or move) inverts the orientation of the respective element.

Consider an alternating cassette with five elements and *m*≥5 bp, and recombination events that do not alter the size of the cassette (i.e., inversions). First note that red blocks in position three and five can move into the first position via a single recombination event (Fig. 2f). Furthermore, a red block in position one can be inverted by first moving to position three, then to five, and back again. A straight-forward recipe to create an arbitrary code made of a single red block is then to: i) move the block into the first position (if required); ii) change its orientation (if required); and finally iii) excise the remaining blocks.

Similarly, to generate an arbitrary code composed of a red and a green block from an alternating cassette with six elements, we can perform steps i) and ii). Then we apply the same procedure to the green blocks, leaving the first block untouched. This results in the first two blocks of the cassette being identical to the desired code. To generate the size-stable code, elements that are not part of the code are excised.

Finally, for a cassette with seven elements, sequentially following the recipe given above, the first three blocks can be populated such that they match any possible code before excising the remaining blocks. This shows that any possible code of size one to three can be created via Lox recombination if the cassette is alternating, *n*≥7, *m*≥5 bp, and flanking sites are inverted.

Under constitutive Cre expression, barcodes with three elements can still undergo inversions via the flanking sites, which reduces their code diversity by a factor of two. The code diversity is therefore that given in Eq. (1).

### Design of code element sequences

That barcodes generated from a Lox cassette are pre-defined in terms of sequence and position in the genome represents an advantage over existing in situ barcoding systems that rely on insertion site analysis for barcode readout [32, 49]. If codes-reading was error-free, choosing code elements of a particular color (red, green or blue, see Fig. 2d for the definition) from a set of sequences that differ at least by one bp pair in both orientations would be sufficient. The maximum number of such elements is easily computed as (4^*m*^−4^*m*/2^)/2 and 4^*m*^/2 for *m* even or odd, respectively, which is large even for small *m*.

**Fig. 3:**
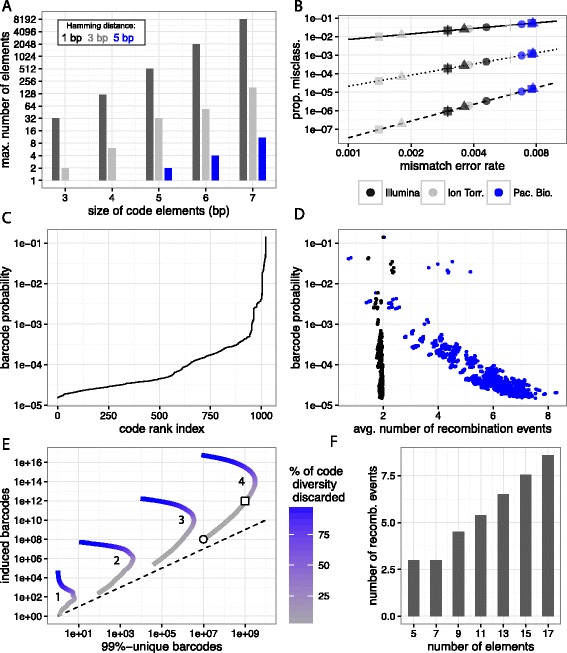
Design of code elements and probabilistic features of optimal Lox cassettes. **a** Numerically determined maximal size of sets of elements that are separated by a minimal Hamming distance of 1 bp (*black*), 3 bp (*gray*), and 5 bp (*blue*). In order to be robust to two sequencing errors, the minimal distance needs to be 5 bp, which requires *m* to be larger than 4 bp. **b** Upper bound for the expected proportion of misclassified elements as a function of empirical DNA sequencing mismatch error rates [52] for three common sequencing platforms (Illumina, Ion Torrent, Pacific Biosciences) and different sequence data (P. falciparum (∙), E. coli ($\blacktriangle $), R. spha. (■), H. sapiens (+)). The minimal distance that separates the elements is 1 bp (*solid*), 3 bp (*dotted*), and 5 bp (*dashed*). **c** Ranked probabilities of the 1022 size-stable barcodes from a cassette with 13 elements generated under constitutive Cre expression. While a few codes are relatively frequent, the majority of codes are rare. **d** Scatter-plot showing barcode probabilities against the average number of excisions (*black*) and the number of inversions (*blue*) that are needed to generate from an initially 13 element optimal cassette a size-stable barcode. **e** Maximum number of cells in which a barcode can be induced versus the number of cells that produce 99 %-unique codes, for one to four sequential cassettes. The color represents the percentage of discarded codes relative to the total code diversity, which can be adjusted to experimental conditions post acquisition. Inducing barcodes in a target population of 10^8^ (*circle*) and 10^12^ (*square*) cells yields a proportion of 10 % and 0.1 % of 99 %-unique barcodes respectively. **f** Although code diversity grows as *O*(*n* ^3^), the expected number of recombination events that are needed to generate a size-stable code increases linearly with the number of elements in the initial cassette

With reading errors, in order to remain perfectly robust to one mismatch error, elements of a given color need to differ by at least three base pairs in both orientations for nearest-neighbor matching to be able to correct the error [50]. To ensure correction of *j* mismatch errors, the minimal required Hamming distance between the code elements is 2*j*+1 bp. The size of the sets of elements that meet this condition quickly decreases with increasing *j* (see Fig. 3a for numerical estimates). To reliably be able to correct for two sequencing errors requires *m*≥5 bp.

Assuming that sequencing errors arise independently and error rates are identical for all bases, the number of mismatch sequencing errors in a code element of size *m* is Binomial with parameters *m* and the error probability per bp [51]. Any element that has *j* or less errors will be classified correctly by nearest-neighbor matching. The probability of more than *j* errors gives an upper bound for the expected proportion of misclassified code elements. Fig. 3b shows this for elements of size *m*=7 bp as a function of the minimal distance and the mismatch error rates for next-generation sequencing platforms [52]. Different symbols indicate different sequence data. Even for low-fidelity platforms like Pacific Bioscience single molecule real time sequencing, a minimal distance of five bp results in less than ten misclassified elements per million.

A concrete example for an alternating Lox barcoding cassette with 13 code elements of size seven bp each (in bold), and robust to two sequencing errors per element (i.e. the minimal Hamming distance between elements of the same color is 5), is:

ATAACTTCGTATA ATGTATGC TATACGAAGTTAT **AAAAAAC**ATAACTTCGTATA GCATACAT TATACGAAGTTAT **AAACCCG**ATAACTTCGTATA ATGTATGC TATACGAAGTTAT **AACGCTA**ATAACTTCGTATA GCATACAT TATACGAAGTTAT **AGTCATC**ATAACTTCGTATA ATGTATGC TATACGAAGTTAT **ACCCGCC**ATAACTTCGTATA GCATACAT TATACGAAGTTAT **ATGAACA**ATAACTTCGTATA ATGTATGC TATACGAAGTTAT **ACGTTAA**ATAACTTCGTATA GCATACAT TATACGAAGTTAT **CACTGAA**ATAACTTCGTATA ATGTATGC TATACGAAGTTAT **CAACTGA**ATAACTTCGTATA GCATACAT TATACGAAGTTAT **CCAATCC**ATAACTTCGTATA ATGTATGC TATACGAAGTTAT **CCTCCAG**ATAACTTCGTATA GCATACAT TATACGAAGTTAT **GCCCCGA**ATAACTTCGTATA ATGTATGC TATACGAAGTTAT **CGTAGCA**ATAACTTCGTATA GCATACAT TATACGAAGTTAT.

### Probabilistic features of optimal Lox cassettes

In this section we explore probabilistic features of the optimal design: the probability to generate each of the final codes; and the number of recombination events that are needed to create size-stable codes. For the analysis, we make two assumptions: first, all interactions with Lox sites that are at least 82 bp apart are equally likely; second, recombination events occur sequentially and independently.

#### Barcode distribution is heterogeneous

Size-stable barcodes of a Lox cassette are randomly generated and not all codes are equally likely. This is in contrast to the Rci invertase based approach implemented by Peikon et al. [35], who reported a close to the ideal uniform distribution of barcodes generated in E. coli. after several recombination events.

Although an analytical expression for the probability mass function of final codes is not available, stochastic simulations enable us to study properties of practical importance such as the probability of generating a code more than once. Ensuring this probability is low is important in practice because progeny of two cells that independently generate the same code will be confounded as pertaining to the same clone.

Figure 3c shows the probability to be generated for each of the 1022 codes that ensue from a cassette with 13 elements (sorted in ascending order). To produce this plot, 10^8^ barcodes were Monte Carlo generated in silico via sequential recombination of the initial cassette. The number of times a specific code appeared was recorded, normalized and sorted. While some codes are relatively frequent, most are rare. In Fig. 3d, the average number of recombination events (inversions: blue, excision: black) is plotted as a function of barcode probability. The number of inversions and barcode probability are negatively correlated, an indication that rare codes undergo, on average, more inversions. The number of excisions is close to two for all codes.

Ideally, each cell is tagged with a unique barcode. As with all existing barcoding techniques however, 100 % unique barcodes cannot be guaranteed unless each cell is separately transduced with a different code, an approach pursued by Grosselin et al. [53]. What influences the expected number of unique barcodes is the code diversity *D*, *p*_*i*_, the probability of code *i*, where *i*∈{1,2,…,*D*}, and *j*, the total number of codes that are generated. Using analysis of the generalised birthday party problem [54], the expected proportion of unique codes is

2 $$\begin{array}{*{20}l} \sum_{i=1}^{D}p_{i}(1-p_{i})^{j-1} \approx 1-(j-1)\sum_{i=1}^{D}{p_{i}^{2}}, \end{array} $$

where the numerically convenient approximation on the right hand side arises from a Taylor expansion around 0 and is appropriate if (*j*−1)≪1/(max*ip*_*i*_). Relatively large *p*_*i*_’s negatively affect the expected proportion of unique codes. Therefore, for heterogeneous barcode distributions, a natural strategy is to discard most frequent codes in order to exclude from the analysis barcodes that are more likely to be induced more than once. In the following, we assume that from all induced barcodes, keeping a subset that contains on average 99 % unique barcodes is sufficient for most applications and call these barcode sets 99 %-unique.

Using the approximation Eq. (2), in Fig. 3e we computed the maximum number of cells in which a barcode is induced versus the number of induced barcodes that are 99 %-unique, for one to four sequential cassettes (indicated by the numbers 1 to 4). The color represents the percentage of discarded codes relative to the total code diversity. This parameter can be adjusted to meet the specific needs of a given experiment. For instance, for four concatenated cassettes with 13 elements each, inducing barcodes in a target population of 10^8^ cells yields 10 %, or 10^7^ 99 %-unique barcodes (indicated by a circle). If the target population is larger, e.g., 10^12^ cells (indicated by a square), the proportion of 99 %-unique to total induced barcodes is reduced (approximately 0.1 %), giving 10^9^ single cells that carry a 99 %-unique barcode.

These results show that by discarding frequent codes from the read-out, large numbers of clones can be tracked with high confidence, suggesting Cre Lox in situ barcoding is suitable for high-throughput lineage tracing experiments.

#### Number of recombination events to generate barcodes does not diverge with cassette size

If Cre is expressed for long enough, Lox cassettes will eventually become size-stable. The time this will take correlates with the number of recombination events that separate a stable barcode from its initial cassette. Below, we estimate this quantity using the theory of absorbing Markov chains.

In a cassette with *n* elements, there are *n*+1 Lox sites. The number of Lox pairs that are flanking *k* elements is *n*+1−*k*. Lox pairs that have less than three elements in between do not interact, as they are separated by less than the minimal 82 bp distance. Pairs of Lox sites that have three or more elements in between are termed productive. For *n*≥3 the number of productive pairs is $\sum _{k=3}^{n}(n+1-k)=(n-1)(n-2)/2$, and the number of productive pairs, where recombination leads to excision, i.e. where an even number of elements separates the two sites, is

$$\begin{array}{*{20}l} \sum_{3\leq k\leq n: k~\text{even}}(n+1-k)={\frac{(n-1)(n-3)}{4}} \end{array} $$

for *n* odd. The equalities are a direct consequence of evaluating the respective sums. The probability that a productive pair excises exactly *k* elements is given by the ratio of productive pairs that are separated by *k* elements by the total number of productive pairs, i.e.

3 $${} \begin{aligned} P(\textrm{excision of }k~\text{elements})&=\frac{n+1-k}{\sum_{k=3}^{n}(n+1-k)}\\ &= \frac{2(n+1-k)}{(n-1)(n-2)}, \end{aligned}  $$

for *k* even, 3≤*k*≤*n*, otherwise it is zero. Similarly, the number of productive pairs where recombination leads to inversion is (for *n* is odd)

4 $$\begin{array}{*{20}l} \sum_{3\leq k\leq n,k~\text{odd}}(n+1-k)={\frac{(n-1)^{2}}{4}}, \end{array} $$

and the probability that interaction of a productive pair leads to an inversion is

5 $$\begin{array}{*{20}l} P(\text{inversion})=\frac{2(n-1)^{2}}{4(n-1)(n-2)}={\frac{n-1}{2(n-2)}}. \end{array} $$

Equations (3)–(5) enable a description of the formation of size-stable barcodes as a discrete-time absorbing Markov chain. The number of elements in the cassette corresponds to its state, and Eqs. (3) and (5) give the transition probabilities from *n* to *n*−*k*, and from *n* to *n* elements respectively. There are *n*−3 transient and 4 absorbing states. Absorbing states are cassettes that have either three, two, one, or zero elements. Absorbing Markov models are well understood, and a wealth of theoretical predictions regarding their properties are available [55]. These include the average number of steps until reaching an absorbing state, starting in one of the transient states. The fundamental matrix of this Markov Chain is

$$\begin{array}{*{20}l} N= (I_{n-3}-Q)^{-1}, \end{array} $$

where *I*_*n*−3_ is an (*n*−3)×(*n*−3) identity matrix, and *Q* is the transition matrix corresponding to the transient states. The expected number of recombination events, starting with a cassette of *n* elements, until reaching a final code is then the *n*^th^ entry of the vector *t*=*Nc*, where c is a column vector all of whose entries are 1.

In Fig. 3f, the average number of recombination events that separate the initial cassette from a final code is shown as a function of the cassette length. Although code diversity grows as *O*(*n*^3^), the number of recombination events required to generate a code increases linearly in *n*.

## Discussion

***Lox barcode cassettes with code elements of size four***

When we derive the upper code diversity bound and the optimal Lox barcode cassette, we focus on code elements in the regime 5 bp≤*m*<24 bp. These have maximal size-stable barcodes of three elements that are largely insensitive to over and under estimation of the minimal Lox interaction distance. For *m*<5 bp, size-stable barcodes of four elements are possible and their maximal code diversity grows as *O*(*n*^4^). These are stable, however, only if the minimal interaction distance between two Lox sites is greater than 80 bp, a distance at which interactions have shown to still be possible in vivo in the similar Flp/FRT system [39].

**Fig. 4:**
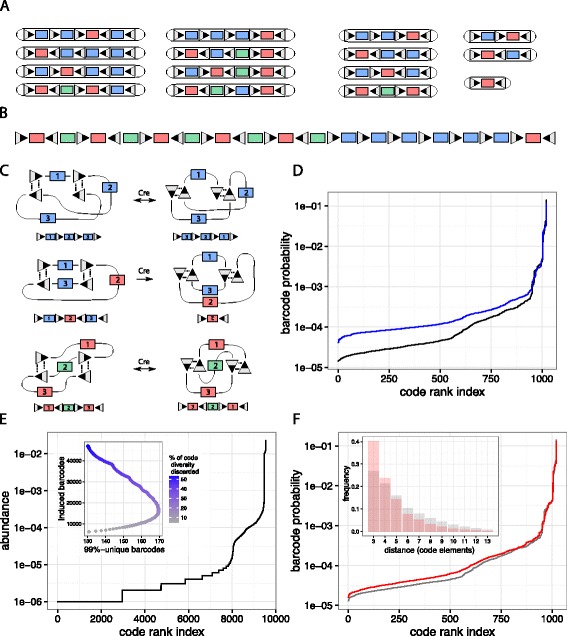
Short code elements, higher order Lox interactions, transient Cre activation, and distance dependent Lox-Lox complex formation. **a** Possible size-stable barcodes if *m*<5 bp. **b** Cassette with 17 elements and *m*=4 bp that attains an effective code diversity of 19,716 barcodes, requiring that the minimal Lox interaction distance is greater than 80 bp. **c** Higher order Lox interactions, where two or more pairs of Lox sites recombine simultaneously, can lead to unexpected recombination products. **d** Estimated barcode distribution if up to two Lox pairs can interact simultaneously (*blue*). The distribution becomes flatter at the lower end, which implies that rare codes are more likely compared to a scenario in which recombination events occur sequentially only (*black*). **e** Mimicking a short Cre activation pulse in a population of a million cells carrying a 13 element Lox cassette, the number of recombination events is assumed Poisson distributed with mean 1. The main graph shows code abundance after the pulse, where almost 10^4^ distinct barcodes are generated, with circa 30 % being generated only once. The inset, similar to Fig. 3 e, gives the number of 99 %-unique barcodes as a function of induced and discarded barcodes. **f** Distance dependent Lox-Lox interactions. Assuming that the likelihood for two productive Lox sites to form a complex is inversely proportional to their distance, interactions between sites that are close-by are favored over interactions of sites that lie further apart (see inset for the distribution over distance dependent (*red*) and uniform (*gray*) Lox-Lox interactions). The difference in barcode probabilities is relatively small between the distance dependent (*red*) and uniform (*gray*) scenario, their distribution being more homogeneous if close-by sites form complexes more frequently

Most interesting is the case *m*=4 bp, which permits correction of one sequencing error with six code elements that are 3 bp apart in both orientations (see gray bars in Fig. 3a). The upper diversity bound is derived along the same lines as for *m*≥5 bp (see Fig. 4a for possible stable codes), which gives

$$\begin{array}{*{20}l}{} 48{{n_{r}}\choose{1}}{{n_{b}}\choose{3}} &+64{{n_{r}}\choose{2}}{{n_{g}}\choose{1}}{{n_{b}}\choose{1}} +12{{n_{r}}\choose{1}}{{n_{b}}\choose{2}}+\\ &+16{{n_{r}}\choose{2}}{{n_{g}}\choose{1}} +4{{n_{r}}\choose{1}}{{n_{b}}\choose{1}} +2{{n_{r}}\choose{1}}. \end{array} $$

To maximize usage of the 6 code elements, we start with a cassette that has six red, five green and six blue blocks, i.e. {*n*_*r*_,*n*_*g*_,*n*_*b*_}={6,5,6}. This gives an upper diversity bound of 36996 barcodes. As confirmed by Monte Carlo simulations, this upper bound is attained by a cassette with inverted flanking sites in which the first 11 Lox sites are alternating, and the remaining sites, except the last, are oriented in the same direction as the first Lox site (Fig. 4b). Under constitutive Cre expression, barcodes with four elements can still undergo inversions, and the effective code diversity is 19,716.

Careful measurements will be needed to determine whether Lox sites at a distance of 80 bp still interact. If they don’t, the cassette shown in Fig. 4b with *m*=4 bp represents an interesting alternative to the barcode cassette design described in the main text, as with less elements it reaches higher code diversity, but at the cost of less robustness to sequencing error and hence barcode readout fidelity.

***Higher order Lox interactions***

In the Cre Lox system, single recombination events always involve exactly two Lox sites. However nothing except DNA flexibility prevents several pairs of Lox sites to interact at the same time. The rate at which pairs of Lox sites bind simultaneously depends on the number of Lox sites and the kinetic rates of Lox-Lox complexes. In vitro, the latter appear surprisingly stable [40] and together with the potentially large number of Lox sites in the barcode cassettes, make simultaneous interactions a plausible possibility.

Higher order Lox interactions lead to unexpected and in certain cases novel recombination products (Fig. 4c). For example, simultaneous interactions of two overlapping pairs of Lox sites oriented in the same direction do not result in excision, but in a reordering of the sequences between the sites. Similarly, if pairs are inverted, simultaneous recombinations do not invert but excise the sequence between the outermost sites.

For the alternating cassette and *n*≥7, multiple concurrent Lox interactions do not generate additional codes as the upper code diversity bound is already attained. Therefore our results on Lox barcode design and code elements remain unchanged in the presence of higher order Lox interactions. What does change is the distribution over barcodes, which flattens in the tail if more than one Lox pair recombines at a time (Fig. 4d).

***Transient Cre expression***

Code diversity strongly depends on the number of elements in size-stable barcodes. If Cre is expressed constitutively, size-stable barcodes with code elements of size *m*≥5 bp have a maximum of three elements. One possibility is to create transient Cre activity rather than constitutive.

A well tested system that provides temporal control over Cre activity is tamoxifen inducible CreEr [42]. In the presence of tamoxifen, the fusion protein CreEr, which is normally located in the cytoplasm, is transported into the nucleus, where it can bind to Lox sites and induce recombination. Depending on the duration of Cre activation and its efficiency, stable sequences with more than three elements are likely to be generated from a Lox barcode cassette. Although most of these sequences are stable only in the absence of Cre, in this section we make no distinction between these and the size-stable barcodes defined earlier.

Figure 4e shows barcode probabilities after activation of CreEr in 10^6^ cells with an optimal Lox cassette of size 13. The number of recombination events induced by transient CreEr activity is assumed Poisson distributed with mean one. About 10^4^ distinct barcodes are generated, and 30 % of these appear only once. For comparison, the inset, similar to Fig. 3e, indicates that a maximum of circa 170 99 %-unique barcodes are generated from a single cassette, by inducing barcodes in about 17000 cells. For a Poisson distributed number of recombination events with mean one, this is 30 times more than what is feasible with size-stable codes from the same cassette.

Although highly promising in terms of code diversity, it should be noted that potential drawbacks of this approach are the length of the barcodes (leading to more involved code sequencing), leakiness of CreEr into the nucleus in non-induced cells [56], the relatively long half-life of tamoxifen [57], and a barcode probability that depends on the efficiency of Cre induction.

***Distance dependent Lox-Lox complex formation.***

Cre and co-localization are necessary for two Lox sites to form a complex. Therefore the distance between two sites, in addition to the minimal distance constraint considered so far, is likely to impact on Lox-Lox recombination efficiencies. In this section we analyze how distance dependent Lox-Lox interactions change barcode probabilities relative to uniform interactions.

Modelling DNA as a flexible polymer, the probability of a Lox-Lox complex is predicted to be inversely proportional to the distance in bp between sites [58]. Together with a minimal distance of 82 bp, we use this model to compare the distribution over barcodes with the distance-independent scenario for the 13-element optimal Lox barcoding cassette. As shown in Fig. 4f, Lox sites that are closer form complexes more often relative to the uniform case (inset), and barcode probabilities are more homogeneous. Thus, in our model, distance dependent Lox-Lox complex complex formation improves mixing of Lox barcode cassettes before reaching their size-stable configuration.

## Conclusions

Existing cellular barcoding approaches have already lead to significant biological discoveries and so new approaches that overcome their shortcomings are inherently desirable. Here we have established that using Cre Lox, it would be feasible to create an in situ, triggerable barcoding system with sufficient diversity to label a whole mouse, and propose this as a system for experimental implementation.

## Methods

Simulations for predicting barcode probabilities and computing the number of 99 %-unique barcodes (Figs. 3c–e and 4d–f) are implemented in custom C++ code and visualized in R.

## Abbreviations

bp, base pair; DOX, doxycycline; kb, kilobase

## Additional file

Additional file 1 The C++ code provided in ‘Additional file 1’ computes barcode probabilities (Fig. 3 c) and average number of inversions and excisions (Fig. 3 d) for a Lox barcoding cassette with m Lox sites. With modifications (specified at the end of the file) it also computes the data for Figs. 3 e and 4 e (inset) and distributions shown in Fig. 4 d-f. CPP 12.1 kb
